# Retrospective Analysis of Clinicopathological Characteristics of Surgically Treated Basal Cell Carcinomas of the Face: A Single-Centre Maxillofacial Surgery Experience

**DOI:** 10.3390/jcm13185470

**Published:** 2024-09-14

**Authors:** Abdullah Saeidi, Aydin Gülses, Maryam Jamil, Albraa Alolayan, Shadia Elsayed, Jörg Wiltfang, Christian Flörke

**Affiliations:** 1 Department of Oral and Maxillofacial Diagnostic Sciences, College of Dentistry, Taibah University, Madinah 42353, Saudi Arabiassayed@taibahu.edu.sa (S.E.); 2 Department of Oral and Maxillofacial Surgery, University Medical Center Schleswig-Holstein, Campus Kiel, 24118 Kiel, Germany; 3 King Fahd General Hospital, Jeddah MOH, Jeddah 21589, Saudi Arabia; maryajamil@gmail.com

**Keywords:** nonmelanoma skin cancer, basal cell carcinoma, recurrence, histopathology, retrospective

## Abstract

**Background**: Basal cell carcinoma is the most common nonmelanoma skin cancer, followed by cutaneous squamous cell carcinoma. The objective of the current study was to retrospectively evaluate the epidemiology, characteristic variations, histological aspects, and prognosis of basal cell carcinoma of the facial region based on a single-centre experience. **Methods**: Data from 125 patients admitted to the Department of Oral and Maxillofacial Surgery, University Medical Center Schleswig-Holstein (UKSH), Kiel, for surgical treatment of basal cell carcinomas of the face between January 2015 and April 2021 were evaluated. **Results**: The mean patient age was 79.58 years, 60.5% were male and 39.5% were female. Six patients (4.8%) had tumour recurrence with no regional metastasis. Seventy-nine patients (63%) were classified as T1. The nose and the temporal region were the most common areas. The mean tumour thickness was 3.20 mm. **Conclusions**: Micronodular, sclerosing/morphoeic, nodular, and superficial growth patterns of basal cell carcinoma are highly correlated to recurrence, so an excision safety margin is recommended. There is a strong correlation between tumour thickness and recurrence among basal cell carcinoma cases. When completely excised, the recurrence rate for basal cell carcinoma is relatively low.

## 1. Introduction

Basal cell carcinoma (BCC) is the most common malignant skin tumour, representing 5% of all skin cancers with increasing incidence worldwide [1]. There is a 30% lifetime risk for the white population of developing BCC [2]. Between 1998 and 2010, German cancer registry data displayed a 2.4-fold increase in basal cell carcinoma. Among German patients with BCC, the 5-year absolute survival was 87.1% and overall BCC mortality rates were low [3,4]. The interaction of environmental, genetic, and phenotypic variables is highlighted by recent pathogenesis findings of BCC [5]. BCC carcinogenesis is significantly influenced by the Hedgehog signalling pathway, specifically by alterations in the PTCH1 gene [6]. UV light increases the expression of matrix metalloproteinases (MMPs), which facilitate invasion and angiogenesis [7]. Because of the significant risk of morbidity and death, along with the impact of the disease on the sufferer, prevention, early detection, and treatment of skin cancer are essential [4]. Diagnosis of BCC usually takes place during a routine skin examination [8]. The classification of BCC is crucial for appropriate treatment and prognosis. Clinically, BCC presents as nodular, superficial, morpheiform, ulcerative nonhealing, pigmented, or fibroepithelioma of Pinkus papule [9]. Histologically, it is classified as superficial, nodular, infiltrative, or morpheiform subtypes [10]. These growth patterns correlate with the risk of incomplete surgical excision and recurrence. High-risk subtypes include infiltrating, morpheiform, and micronodular carcinomas. The Royal College of Pathologists recommends reporting growth patterns, differentiation, and excision margins in histopathological assessments [11].

Basal cell carcinoma is diagnosed by clinical presentation and histopathological evaluation, which includes appearance, anatomic location, morphology, genetic factors, and the patient’s history [12]. Tumour identification has been greatly enhanced by recent developments in BCC diagnosis [13]. Dermoscopy, reflectance confocal microscopy (RCM), and high-frequency ultrasound (HFUS) are examples of non-invasive advanced imaging techniques that have improved tumour assessment and diagnostic accuracy [14]. Dermoscopy has been shown to be especially helpful in identifying particular subtypes [15]. While HFUS aids in determining the size, shape, and tumour thickness, RCM permits tumour-free margins. In addition to optical coherence tomography and optical spectroscopy, these imaging technologies provide earlier and more accurate diagnoses without requiring repeated biopsies [4,16,17]. 

With considerable variations depending on the size and location of the tumour, BCC has low rates of metastasis and recurrence. Morgan et al. (2020) found that for large BCCs (≥2 cm), the local recurrence rate was 8.9% and the metastasis/death (M/D) rate was 6.5%, while the M/D rates for small BCCs were 0.8% and 0%, respectively [18]. According to Granger et al. (2023), high-risk BCCs have a recurrence risk of roughly 5% after five years, which rises to 12% after ten [19]. Higher rates were linked to the male gender and head location.

The primary goal of the current study was to evaluate retrospectively the epidemiology, characteristic variations, histological aspects, and prognosis of basal cell carcinoma of the facial region based on a single-centre experience in northern Germany to provide readers with potentially new insights and correlations.

## 2. Materials and Methods

This retrospective study was approved by the local ethical review committee of Christian-Albrechts-University, Faculty of Medicine (D 430/21. 22 Febuary 2021). Patients admitted to the Department of Oral and Maxillofacial Surgery, University Medical Center Schleswig-Holstein (UKSH), Kiel, for treatment of skin malignancies involving the head and face region between January 2015 and April 2021 were included. Data collection was performed via the ORBIS Information Management System (ORBIS AG, Saarbrücken, Germany). Cases were screened using specific diagnostic codes for head and neck skin cancer, including C44 01, C44 310, C44 311, C44 319, and C44 91.

### 2.1. Study Design

In our study, the end of observation was determined by death, the occurrence of a tumour recurrence (real observation time), or the end of the observation time (censored observation time). No distinction was made here between death from a tumour or death from another event. 

The surgical operation was standardised in our department starting with a circular excision with at least a 5 mm safe margin around the lesion. The main lesion excision was marked with a non-resorbable suture material in at least three regions to help the pathologist orientate the histological specimen with the clinical situation. The histopathological specimen was sent in a formalin container to the laboratory with a detailed report of all the clinical and pathological data. Directly after the first procedure, the surgeon divided the skin where the lesion was into four areas. Five biopsies were taken, including the four borders of the lesion and the floor of the already removed lesion. The five biopsies were placed separately in labelled containers and sent to the pathology department for an immediate and definitive histological examination.

The skin defect was covered with a dermal temporary coverage template and sutured to the wound borders. The first operation was performed after ensuring that there was no bleeding and that the patient’s condition was stable. Two to three days later, after receiving the histopathological reports from the pathology department, the patient was recalled. If there was tumour residue, the relevant region was removed and sent again to the laboratory for a second evaluation. After receiving final confirmation from the pathologist that the lesion was completely removed, the final defect reconstruction plan was discussed with the patient, after explaining the advantages and disadvantages of the possible reconstruction techniques in this region of the face. 

According to the classification standards of the Union for International Cancer Control (UICC), the information on the TNM status was taken from the histopathological report, as well as information on the tumour thickness and the resection status. Histopathological and physician surgical reports were used to generate information on tumour histopathological growth type, tumour thickness, tumour localisation, and surgical techniques.

Patient follow-up medical records had to be available for at least 3 years. The time of tumour recurrences (local recurrences, lymph node recurrences, and distant metastases) and secondary tumours were recorded as part of follow-up examinations. Disease-free survival (DFS) was determined, and all information was anonymised and transferred to an Excel database.

This study includes the 125 patients who were treated for basal cell carcinomas (BCCs) in the Department of Oral and Maxillofacial Surgery, University Clinic Schleswig Holstein, Campus Kiel. 

The following inclusion criteria were specified. (1) Histological confirmation that the patient had at least one primary nonmelanoma skin cancer (especially basal cell carcinoma) based on biopsy results and a referral from the dermatology department. (2) The primary tumour should have been treated surgically only. (3) The location of the skin lesion must be limited to the face. (4) An initial diagnosis must have been made between the years 2015 and 2021. (5) A follow-up period of at least 3 years must be included. 

Exclusion criteria were the following. Patients who showed incomplete data such as histopathological, therapeutic, or operative records were excluded. (1) Patients who were treated primarily with non-surgical therapy (palliative, radiation, or chemotherapy). (2) Incomplete/insufficient therapy or death documentation. (3) Another head and neck malignancy. (4) Patients with more than one cancer were observed and reviewed separately through the ORBIS information management system and medical record recurrences. Metastasis or secondary cancers were followed up for several months between the therapy and their reoccurrence. 

The following anamnestic and epidemiological parameters were collected from the digitised medical records in an Excel spreadsheet: (1) age, (2) gender, (3) cancer residual or recurrence, (4) tumour metastasis, (5) T-classification, (6) localisation, (7) histological growth type, and (8) tumour depth. In our study, the tumour size (T-classification), tumour depth, and growth type were extracted from the histopathological reports for each patient. The histopathological reports were written by our colleagues in the pathology department after they studied each case accordingly. Tumour sizes were classified according to staging manuals of the American Joint Committee on Cancers (AJCC) and the 8th edition UICC and TNM classification. The tumour diameter used as a distinguishing factor was classified as follows: T1 size ≤ 2 cm; T2 size > 2 cm and <4 cm; T3 tumour diameter ≥ 4 cm or minor bone erosion or perineural invasion 0.1 mm in caliber or deep invasion ≥ 6 mm beyond the subcutaneous fat; and T4 with major bone involvement such as skull base invasion, gross cortical bone, or marrow.

### 2.2. Statistical Analysis

Regarding the statistical analysis, the aggregated Excel spreadsheet was transferred to the statistical package for social studies (SPSS 25.0-IBM SPSS Inc., Chicago, IL, USA). Frequency tables, crosstabs, bar and pie charts, and histograms were used to present descriptive statistics. Correlation between different characteristics of a patient was determined using cross tables. Descriptive statistical analysis was performed for all variables, means and standard deviations were calculated for continuous variables, while frequencies and percentages were calculated for categorical data. The probability of the correlations was checked using the chi-square test. A *p* value ≤ 0.05 was considered statistically significant for all statistical tests. Mean differences between the two independent groups were determined with the *t*-test. Correlations among all variables were calculated.

## 3. Results

After applying the inclusion and exclusion criteria, 125 patients with basal cell carcinoma were included, with a mean age of 79.58 years (range 34–107, and median 73). Of the patients, 60.5% were male and 39.5% were female. The recurrence of BCC among the patients was 4.8% (n = 6), and there were no metastases among them. 

**Figure 1:**
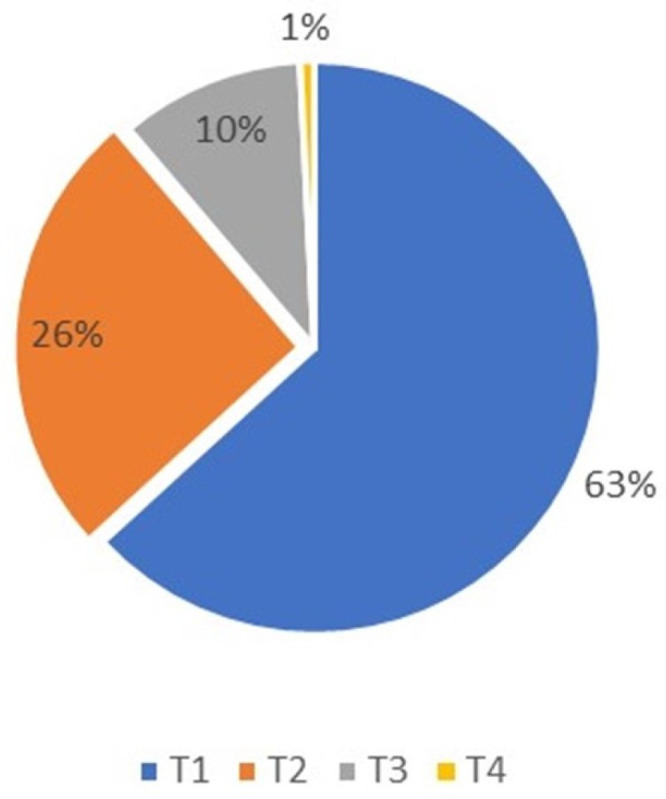
Basal cell carcinoma T-classification distribution, T4 was only 1% and T1 was predominant at 63% of patients.

The T-classification among the patients was as follows: (1) 79 patients (63%) were classified as T1; (2) 32 (26%) as T2; (3) 13 (10%) as T3; and (4) 1 as T4 (Figure 1).

The chi-square test indicated no significant correlation between T-classification and recurrence among patients diagnosed with BCC at a level of alpha 0.05 (*p* = 0.499).

**Figure 2:**
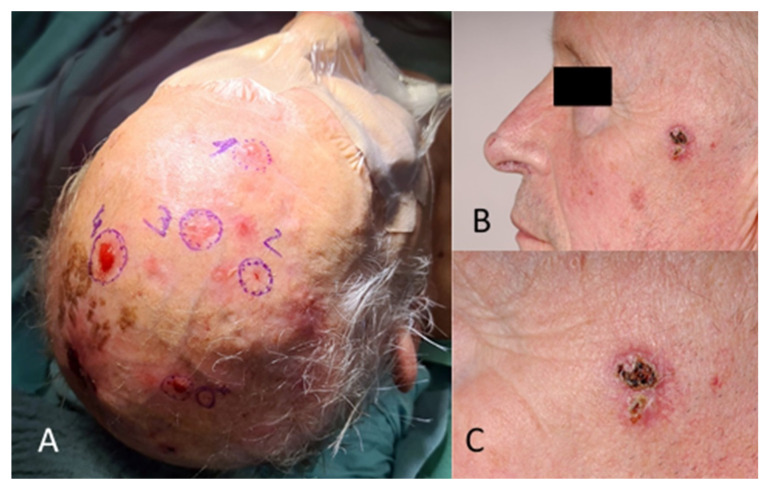
(**A**) Multiple BCC in the scalp and forehead region. (**B**) Different patient with BCC in the temporal region. (**C**) The image in (**B**), magnified.

**Figure 3:**
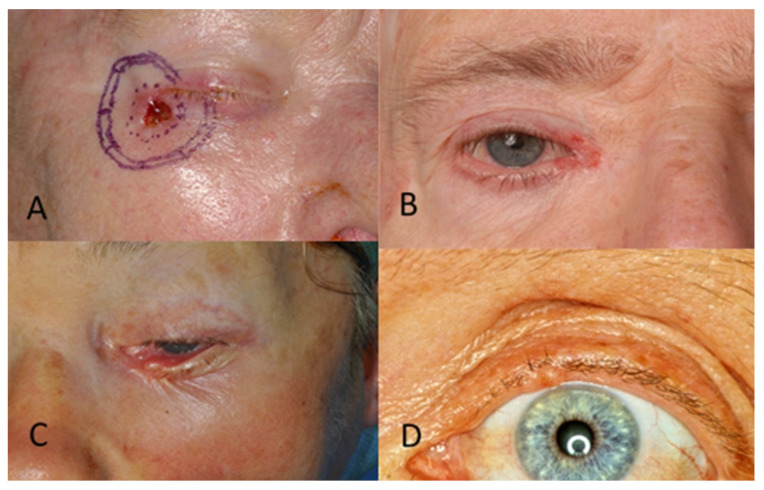
(**A**) BCC in the lateral eye corner. (**B**) BCC in the medial eye corner. (**C**) BCC in the lower eyelid. (**D**) BCC in the upper eyelid.

**Figure 4:**
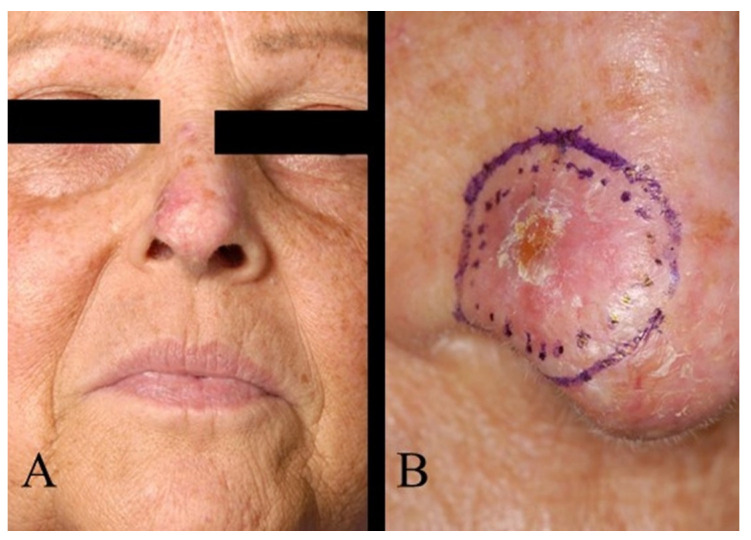
(**A**) BCC of the nose. (**B**) The same patient as in (**A**), magnified image.

**Figure 5:**
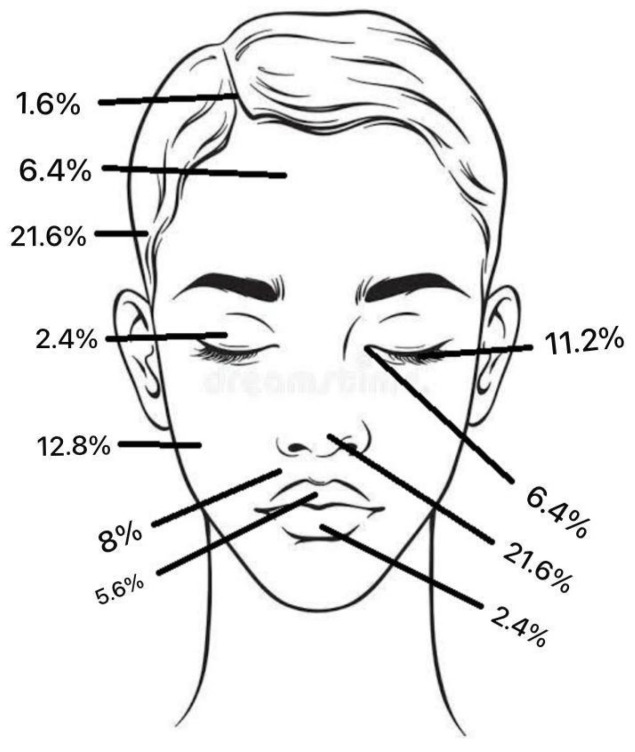
The total frequency % of BCC tumour location.

Regarding tumour location on the face, two patients (1.6%) had BCC in the scalp region, eight (6.4%) in the forehead region, and no patients had BCC in the eyebrow region. In the temporal region, 27 (21.6%) had BCC and, in the cheek region, 16 (12.8%) (Figure 2). In the upper lid region, 3 (2.4%) and, in the lower lid region, 14 (11.2%) had BCC. Eight patients (6.4%) had BCC in the medial eye corner region, and none had BCC in the lateral eye corner (Figure 3). In the nose region, 27 (21.6%) and, in the nasolabial region, 10 (8%) had BCC (Figure 4). Seven patients (5.6%) had BCC in the upper lip, and three patients (2.4%) had BCC in the lower lip (Figure 5). The Pearson chi-square test showed no significant correlation between tumour location and recurrence among BCC-diagnosed patients (*p* = 0.138).

**Figure 6:**
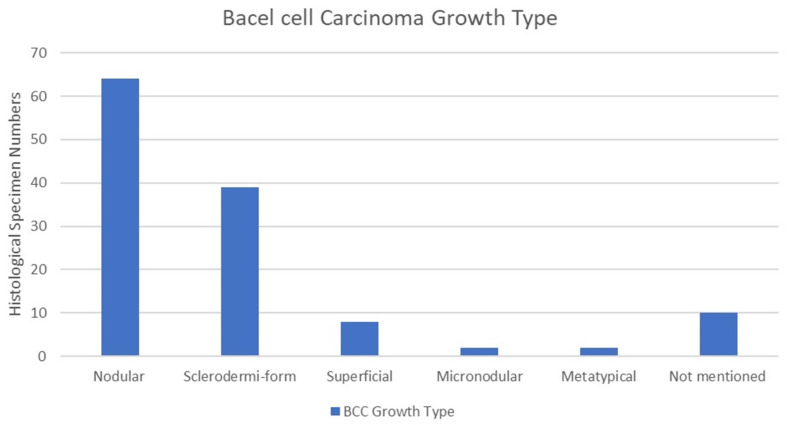
Growth type of BCC. Nodular growth type was the most common among 64 cases, followed by sclerodermiform and superficial growth types, with 31% and 6%, respectively.

Basal cell carcinoma growth type was extracted from the patient’s histopathological report. Nodular growth type was the most common, with 64 cases (51%), followed by the sclerodermiform in 39 cases (31%). The superficial growth type was observed in eight cases (6%), the micronodular growth type in two cases (1.6%), and the metatypical growth type in two cases (1.6%). Growth types were not mentioned in the histopathological reports of 10 cases (8%). It is also important to note that 6 cases out of 125 had tumour recurrence. Among these, two cases were diagnosed as nodular, two as micronodular, one as sclerodermiform, and one as superficial. The chi-square test indicated no statistically significant correlation between growth type and recurrence among BCC patients (*p* = 0.674) (Figure 6).

**Figure 7:**
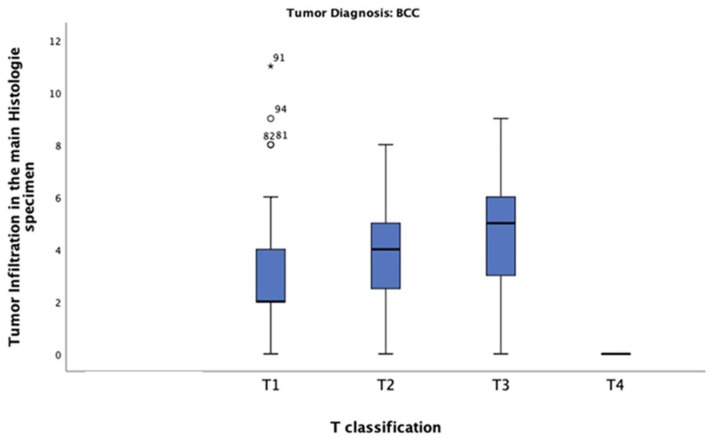
Tumour thickness distribution across T-classification among patients diagnosed with BCC. Most of these patients had a tumour thickness of 2 mm and were classified as T1.

In this study of patients diagnosed with BCC, the mean tumour thickness was 3.20 mm. The highest tumour thickness of 10 mm was observed in one patient only, classified as T4. The most common thickness was 2 mm, observed in tumours classified as T1 (n = 27) (Figure 7). The chi-square test revealed a significant correlation between tumour thickness and BCC T-classification (*p* = 0.0001). 

**Figure 8:**
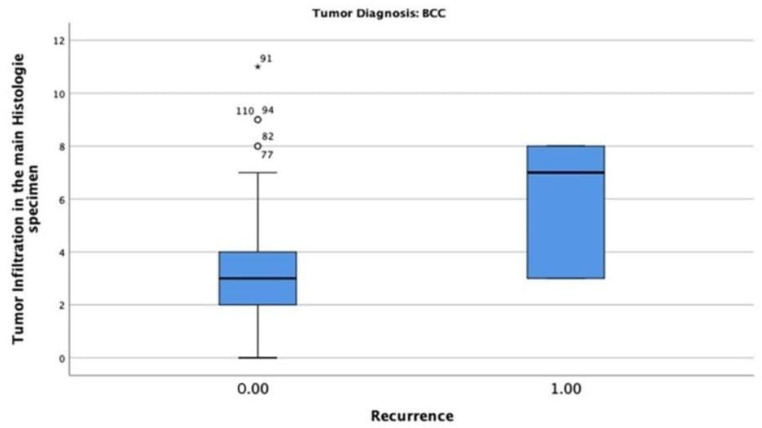
Tumour thickness mean and recurrence rate among patients diagnosed with BCC. Only 6 patients (4.8%) had tumour recurrence with a maximum tumour thickness of 8 mm.

In the BCC-diagnosed patients, only six patients (4.8%) had tumour recurrence, with a maximum tumour thickness of 8 mm. The chi-square test indicated that the correlation between tumour thickness and recurrence was statistically significant (*p* = 0.002) (Figure 8).

## 4. Discussion

To gain and validate insights into the behaviour and treatment of the most common nonmelanoma skin cancer, retrospective data were analysed from 125 patients at the Clinic of Oral and Maxillofacial Surgery in Kiel. The focus of this work was to determine the factors influencing the basal cell carcinoma behaviour of the facial skin. 

The present study has shown that patients older than 60 years of age are more exposed to nonmelanoma BCC, so a routine visit to a dermatologist at this age is highly recommended. BCC affects men more frequently than women. Although the recurrence rate for BCC is relatively low, routine re-examination is still highly recommended. 

Nodular growth was the most common growth type, while micronodular growth was the most uncommon. Micronodular, sclerodermiform, nodular, and superficial growth types are highly correlated to recurrence in BCC, so a further excision safety margin is recommended. When surgically removing BCC, the tumour thickness should be considered. Thickness normally ranges between 0.1 and 7 mm. There is also a strong correlation between thickness and recurrence among BCC cases. The most common sites for BCC on the face are the temporal region, followed by the nose and cheek regions.

Maxillofacial surgeons must understand the importance of considering H (high risk) zones, which include the periorbital, nose, nasolabial, and upper lip regions, M (moderate risk) zones, which include the cheek, lower lip, scalp, and forehead, and L (low risk) zones, which include the neck and jaw lines, for BCCs where more surgical excision of critical structure could impact a patient’s aesthetic outcome. This is recommended even though there is not always a consistent link between H locations and unfavourable histology subtypes [20]. 

The AJCC 8th edition staging system for BCC efficiently detects high-risk cases, although it may overstate certain tumours [18,21,22]. The AJCC manual aims to include molecular criteria for customised staging. However, there is still disagreement over the best treatment plan for superficial BCC based on anatomic sites [23]. Research indicates that, compared to low-risk facial zones, superficial BCCs in high-risk facial zones (H and M) have greater incidences of mixed histology [24]. On the other hand, contradictory data indicates that high-risk facial zones are not associated with poor histology subtypes [25]. Rather, regardless of anatomical location, ulceration is strongly associated with aggressive histological subtypes. The staging system also allows maxillofacial surgeons to be more involved in the early stages of treatment planning, which is critical for managing complex facial BCCs near critical structures like the eyes, nose, and lips. The staging system assists in the planning of surgical approaches that maximise tumour removal while preserving function and aesthetics. One potential disadvantage of the existing staging method is that it is primarily concerned with tumour size, depth, and nodal involvement. However, it may not adequately express the aggressive nature of some histological subtypes [26]. The current findings emphasise the complexities of BCC staging and the need for additional studies to improve risk assessment methodologies and time to first recurrence. Multidisciplinary staging approaches should improve the system’s utility, particularly for BCCs in high-risk facial areas, by integrating histological subtypes and sub-classification based on location and perineural invasion.

Rahimi-Nedjat et al. (2021), during their investigation of 232 patients with nonmelanoma skin cancer, found comparable results with a different study population profile: 69.8% of the patients were male and 30.2% were female. The average age at the first admission in their department was 77.1 years (±11.3 years) [27]. The Institute of Cancer Epidemiology at the University of Luebeck, Germany, observed the incidence of nonmelanoma skin cancer in Germany in the period between 1998 and 2010, focusing on basal cell carcinoma and cutaneous squamous cell carcinoma. They observed that males were more affected than females and that the mean age of all nonmelanoma skin cancer patients was 70 years. They also noted that BCC comprised almost three-quarters of all nonmelanoma skin cancers (71.6%) [3]. Stang et al. (2008), in their national study also found that males were more affected than females and that the average age at the date of admission was 65 years [28].

Savino et al. observed 222 patients with BCC in the eyelid region. This study identified the majority of the patients as T1a (64%), followed by T1b (18%) [21]. In a study of 120 BCC patients, Fidelis et al. found that the majority of patients had T1 classification, and the minority were in T3 and T4 [29]. Demirseren et al. observed 331 BCC cases, 185 of which were described as T1a (55.9%), 143 as T1b (43.2%), and the remaining 3 patients were T2 (0.9%) [30]. These authors also observed tumour recurrence in nine cases, with an overall recurrence rate of 2.7%. All patients classified as T2 had recurrence [30]. In our study, 1 patient out of 13 who was classified as T3 had a recurrence. Recurrence was also observed in small tumours such as T1. Out of 79 patients classified as T1, 4 had BCC recurrence (5%). In the T2 group, only 1 patient out of 32 had a recurrence (3.1%). No significant correlation was found between T-classification and recurrence among tumours diagnosed as BCC. The findings negate the theory that BCC recurrence is related to tumour size, as recurrence was observed in small tumours. 

In this study, the most frequent facial locations for BCC were the temporal region, then the nose region, followed by the cheek, observations which align with those of Kuo et al. [31].

Kiely et al. disclosed in their 2019 study that the most common growth type was the nodular (48.7%), followed by the infiltrative type (22.2%). The micronodular type was the most uncommon (≤1%) [32]. Pampena et al. studied the histopathological pattern of 481 BCCs, finding 51.4% to be nodular, 33.9% superficial, and 14.8% infiltrative [12]. Betti et al. found that the most common types were the nodular (44.2%), followed by the superficial (42.9%) and the infiltrative type (10.6%). The micronodular was the least common type with 1.6% [33]. These data support the theory that the nodular type is the most common amongst the BCC growth types, with the micronodular type the least common. The prevalence of the other types was inconsistent among the different articles. For example, in this study, the sclerodermiform was the second most common type, but this type was not mentioned in any of the other studies. However, other authors have found that the infiltrative and superficial types were more common [29].

Regarding the correlation between growth type and recurrence, Kondo et al. reviewed 116 BCC cases and found that 3 of them had a recurrence, 2 of micronodular type and 1 of sclerodermiform type [34]. In contrast, among 139 BCC cases studied by Takata Pontes et al., 4 showed recurrences, 2 with a superficial growth type, 1 a morpheaform, and 1 a nodular growth type [1]. Chren et al. found that the correlation of histopathological patterns to BCC recurrence rates was not statistically significant [35]. 

Previous research has shown that the depth of a BCC tumour is affected by a variety of factors, including age and sex [36,37]. Lee et al. indicated that the tumour thickness is significantly correlated to the tumour size, and that thickness increases with an increase in the T-classification [38]. Some studies found no correlation between BCC recurrence and tumour thickness [39]. However, Pyne et al. (2020) and Armstrong et al. (2017) found a strong correlation between BCC depth and the risk of recurrence [40,41]. BCC invasion depth varies according to subtype and anatomic site. Wetzel et al. studied 500 samples and found that the mean depth was 0.68 mm, in a range between 0.1 and 5.49 mm. Aggressive BCCs had a higher median dermal invasion (1.04 mm) than non-aggressive forms (0.62 mm) [37]. Another study found micronodular tumours have the highest average depth, followed by infiltrative, nodular, and superficial subtypes [42]. A large-scale investigation showed mean depths ranging from 0.3 mm for superficial BCCs to 1.9 mm for nodulocystic forms, with deeper invasion related to prolonged sun exposure sites [36]. High-frequency ultrasound (HFUS) can be used to quantify BCC depth, with 75 MHz probes showing strong correlation (r = 0.870) with histological measures for thin BCCs (≤1 mm), and 30 MHz probes indicating extremely high correlation (r = 0.951) for thick BCCs (>1 mm) [43]. 

According to maxillofacial surgeons, the location, size, depth, histological subtype, perineural invasion, history of recurrence, patient age, and the general health condition of the patient are the most critical prognostic factors for BCC tumours.

For some BCC low-risk patients, topical medication and non-invasive therapies are good alternatives. According to Morton et al. (2013), photodynamic treatment (PDT) is advised for superficial BCC and some thin nodular BCC [44]. It is considered a safe effective treatment for BCC with low risk [45]. The FDA has approved topical immunotherapy for superficial BCC, which includes creams containing 5% imiquimod and 5% 5-fluorouracil [46]. When surgical procedures are limited due to circumstances such as the COVID-19 pandemic, these non-surgical approaches become especially helpful for individuals with less aggressive lesions, low-risk recurrence, or medical contraindications for surgery [47]. 

Surgical treatment options for facial BCCs include surgical excision, curettage with electrodesiccation, Mohs micrographic surgery, and cryosurgery [48]. Each method has advantages and disadvantages. Mohs surgery is a specific method that is very effective for high-risk cancers. Due to its high cure rates and ability to manage the histological margin, surgical excision is still the preferred treatment for localised BCC. Advanced or metastatic cases have been recently successfully treated with targeted medicines such as vismodegib and sonidegib, which block Hedgehog signalling and may save important organs [13]. Overall, the management of BCC should consider tumour characteristics to optimise outcomes. Individualised care is recommended based on tumour characteristics and patient considerations [49].

According to the retrospective approach, the findings of this study are not suitable for the presentation of evidence since no interviews or examinations of the patients took place. In this study, we tried to counteract the limited significance with datasets characterised by a large number of cases. Despite limitations such as one-centre design and non-standardised documentation (for example, incomplete or missing histological reports or patient comorbidities), we hope that the current study provides insights into the skin malignancies of the face, which yearly affect billions of people worldwide and still pose a great challenge for maxillofacial surgeons. Future studies require complete and standardised documentation to increase data accuracy. 

## 5. Conclusions

The current study aims to improve knowledge in the field of nonmelanoma skin cancer, focusing on demographic data, tumour T-classification, growth type, incidence, tumour thickness, and recurrence. Based on the provided data, the study proposes an algorithm that guides treatment decision-making. 

The nose and the temporal region were the most common BCC facial areas. T1 was the most common T-classification. The mean tumour thickness was 3.20 mm. In cases diagnosed with BCC, micronodular, sclerosing/morphoeic, nodular, and superficial growth patterns were highly correlated to recurrence, so an appropriate excision safety margin is recommended. There was a strong correlation between tumour thickness and recurrence. When completely excised, the recurrence rate for basal cell carcinoma is relatively low.

## Data Availability

The data presented in this study are available on request from the corresponding author.
